# Inequalities in Pediatric Fracture Care Timeline Based on Insurance Type

**DOI:** 10.5435/JAAOSGlobal-D-20-00111

**Published:** 2020-08-04

**Authors:** Brock T. Kitchen, Samuel S. Ornell, Kush N. Shah, William Pipkin, Natalie L. Tips, Grant D. Hogue

**Affiliations:** From the University of Texas Health Science Center at San Antonio, San Antonio, TX (Dr. Kitchen, Mr. Ornell, Dr. Shah, Dr. Pipkin, Ms. Tips, and Dr. Hogue), and the Harvard University, Boston, MA (Dr. Hogue).

## Abstract

**Introduction:**

Socioeconomic and insurance status are often linked with limited access to health care. Despite several government-funded projects aimed at curtailing these barriers, pediatric orthopaedic patients continue to experience delays in receiving timely care for fracture treatments. This delay has been well-identified within the orthopaedic literature but, to our knowledge, has never been characterized based on timeline. Thus, the goal of this study is to evaluate the role of ethnicity, socioeconomic status, and insurance type on the timeline of pediatric patients to obtain orthopaedic care within our community.

**Methods:**

Pediatric patients presenting to our clinic for the treatment of one of 21 most common fractures were included. Patient demographics and the timeline of patient care were collected by retrospective chart review.

**Results:**

Government-funded insurance accounted for 60.6% of the 413 patients. These patients experienced significant (*P* < 0.001) delays in access to care when compared with commercial insurance patients; the time between injury and referral as well as the overall time from injury to orthopaedic evaluation was 2.8 and twofold greater at 4.4 days and 9.2 days, respectively. A strong correlation was established between income levels and insurance type.

**Discussion:**

Pediatric patients with a lower socioeconomic status are more likely to rely on government-funded insurance and experience delays in fracture evaluation.

Substantial efforts are being made to improve access to health insurance for patients of all ages, health, and socioeconomic status within the United States. However, access to health insurance does not always translate to healthcare access. Nearly 2 decades ago, Skaggs et al^1^ demonstrated notable delays experienced by pediatric orthopaedic patients relying on government-funded insurance over commercial insurance in receiving orthopaedic evaluation for the most common fracture types. This trend was observed at both the state and national level.^1,2^ Despite these findings and the advances in public welfare projects, pediatric orthopaedic patients continue to experience a decline in their ability to receive timely fracture care.^3-5^

To our knowledge, no study has elicited the underlying reasons for this disparity, and although this study is not designed to determine the underlying reasons, we hope to better understand the timeline of this disparity within the care cycle. Hence, the aim of this study is to evaluate the effect of ethnicity, socioeconomic status, and insurance type on the timeline of orthopaedic care in our pediatric patient population.

Although it is true that the pediatric orthopaedic patient is often allowed a greater degree of fracture tolerance than adult patients because of remodeling potential and the biology of the young bone, the potential for adverse outcomes cannot be completely dismissed.^1,2,3,4,5,6,7,8,9,10,11,12^ Complications such as malunion or nonunion in pediatric fractures are well described and can result in long-term functional limitations.^9,10^ Thus, the ability for patients to receive prompt orthopaedic care is fundamental to an orthopaedic surgeon's capability to appropriately manage pediatric fractures.

Furthermore, with the rapid emergence of personalized medicine, patient-focused outcome measures are taking a more prominent role in assessing the quality of orthopaedic care and orthopaedic surgeons are becoming more attentive to the factors that contribute to these measured outcomes.^13^ One such factor is the time for a patient to complete all phases of the healthcare cycle, including time to diagnosis, treatment, and recovery. Shore et al^14^ have demonstrated the direct influence of this factor on the quality and value within medicine. Improving the time to diagnosis and treatment may result in superior outcomes and hence increase the value for the patient.

Previous studies investigating access to care for pediatric orthopaedic patients have successfully identified deficiencies in timely access to fracture care; however, none have further characterized these deficiencies by timeline. Thus, the aim of this study is to evaluate the effect of ethnicity, socioeconomic status, and insurance type on the timeline of orthopaedic care in our pediatric patient population visiting our clinic.

## Methods

We performed a retrospective review of patients who presented to our outpatient pediatric orthopaedic clinic between January 1, 2016, and June 30, 2016, with 21 different closed fracture types that occur at a high frequency in pediatric patients (Table 1). All polytrauma patients and fractures that require same day manipulation were excluded because of the emergent nature of those treatments. Demographic and health information regarding patient ethnicity (Caucasian, Hispanic, Black, or other), insurance type (government-funded, commercially-insured, or self-pay), date of injury, date of referral, and date of orthopaedic evaluation was extracted through a review of existing medical records of patients identified using the Current Procedural Technique codes. Our clinic is an academic outpatient clinic not directly associated with a hospital or emergency department. We accept all insurance types and offer no preferential appointments based on insurance type. Data regarding the “date of referral” are electronically received and recorded in the electronic health record. Patients are referred to our clinic from many emergency departments, urgent care centers, and primary care providers throughout the surrounding region. Most insurance providers require a referral before a clinic appointment, and although some of these referral processes can be onerous for patients and clinical staff, substantial effort is made to ask for and obtain appropriate referrals before the scheduled appointment. A large percentage of our patient population was either Caucasian or Hispanic; hence, ethnicity was reclassified into two broad categories, Hispanic and non-Hispanic, for more meaningful statistical analysis. The time between injury and referral, referral and orthopaedic evaluation, and overall time from injury to orthopaedic evaluation was deduced from the date of injury, referral, and clinic visit. Patients were stratified based on their insurance status (government-funded, commercial, or self-pay) and income level, and the average time ranges for each group were calculated. All data were collected with the approval of the Institutional Review Board at University of Texas Health Science Center at San Antonio.

**Table 1:** Breakdown of Fracture Types Used to Identify Patients

CPT Code	Procedure	Number and Percentage of Patients (n)
24500	Midhumerus fracture	5 (1.20%)
24530	Humeral supracondylar fracture	28 (6.74%)
24560	Humeral epicondylar fracture	6 (1.44%)
24650	Radial head/neck fracture	18 (4.33%)
24670	Prox ulna fracture	3 (0.72%)
25560	Radius/ulna shaft fracture	12 (2.89%)
25600	Dist radius/ulna fracture	136 (32.77%)
25622	Navicular fracture	3 (0.72%)
25630	Carpal fracture	8 (1.92%)
26600	Dist radius/ulna fracture	34 (8.19%)
26720	Prox/midfinger shaft fracture	29 (6.98%)
26740	Finger articular fracture	9 (2.16%)
26750	Dist finger fracture	17 (4.09%)
27501	Condylar fracture	1 (0.24%)
27530	Tibial plateau fracture	9 (2.16%)
27750	Tibial shaft fracture	22 (5.30%)
27760	Med malleolus fracture	3 (0.71%)
27786	Dist fibula fracture	32 (7.71%)
27824	Weight bear dist fracture	11 (2.65%)
28470	Metatarsal fracture	24 (5.78%)
28490	Big toe fracture	5 (1.20%)

CPT = Current Procedural Technique

Patients included in the study resided in Bexar and 15 other neighboring counties. Median income for each county was obtained from the Census data published by the US Department of Housing and Urban Development (https://factfinder.census.gov) and used to define the socioeconomic status of patient's families. Median household income for each zip code was collected and compared with the median household income for the respective county to determine the patients' income level as “below median income” or “above median income.” For instance, most of our patients reside in Bexar County (84.9%), where the median annual household income for the fiscal year 2016 was $53,210.^14^ Patients from zip codes within Bexar County with median annual household income less than $53,210 were considered “low income” and vice versa. This method of using zip code-based median incomes is a robust, well-established, widely used metric to determine the income levels for medical studies.^15,16,17,18^ This technique uses a large population, readily available data and zip codes and does not require notable economic investment that other techniques, such as tract-based measures, require.^15,16^

Univariate analysis was performed to identify the differences between variables. A chi-squared analysis was performed to determine an association between ethnicity, income levels, and insurance status. A one-way analysis of variance was performed to determine the differences in the number of days between injury and referral, referral and orthopaedic evaluation, and injury and orthopaedic evaluation based on insurance type (government-funded, commercial, or self-pay) and socioeconomic status. *Post hoc* comparisons (Bonferroni) were calculated when dictated by notable results. Next, a negative binomial regression was used to establish a correlation between insurance status and variables identified as significant in the univariate analyses. A *P* ≤ 0.05 was considered significantly different.

## Results

A Current Procedural Technique code search for evaluation of one the 21 specified fracture types yielded a total of 478 patients. A total of 65 patients were excluded based on the prespecified inclusion/exclusion criteria, such as age older than 18 years, insufficient data, or previously established care by an orthopaedic surgeon. Patients treated on the same day for two related injuries were considered as one incident. Thus, a total of 413 patients were included for the final analysis (Table 2).

**Table 2:** Patient Demographics

Patient Demographics	
Age (mean ± SD), yrs	9.56 ± 4.5
Insurance (n, %)	
Government-funded	250 (60.6%)
Commercial insurance	148 (35.8%)
Uninsured/self-pay	15 (3.6%)
Income (n, %)	
Below median income	204 (49.9%)
Above median income	205 (50.1%)
Race (n, %)	
Caucasian	367 (88.9%)
African American	23 (5.6%)
Native American	2 (0.5%)
Asian/Pacific Islander	8 (1.9%)
Unknown/other	13 (3.1%)
Ethnicity (n, %)	
Hispanic	202 (51.3%)
Non-Hispanic	192 (48.7%)

A chi-squared analysis revealed a significant association between the insurance status and income levels (χ^2^ = 33.228 (Pearson), *P* < 0.001, and Cramer V = 0.290). Patients residing in lower income zip codes were more likely to rely on government-funded insurance (odds ratio = 3.393, 95% CI = 2.264 to 5.393). Similarly, a significant association was observed between ethnicity and income (χ^2^ = 4.307 (Pearson), *P* = 0.038, and Cramer V = 0.105) and insurance type (χ^2^ = 18.079 (Pearson), *P* < 0.001, and Cramer V = 0.214). The Hispanic population was more likely to reside in a lower income zip code (odds ratio = 1.526, 95% CI = 1.023 to 2.276) and rely on government-funded insurance (odds ratio = 2.482, 95% CI = 1.608 to 3.832).

**Figure 1:**
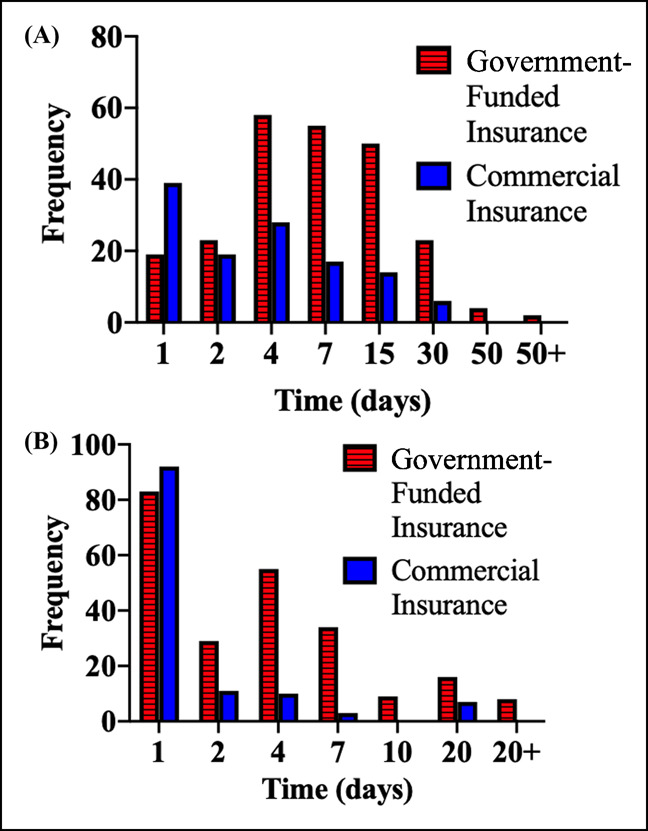
Graph showing the distribution of delay experienced by commercial and government-funded insurance patients: Time between (**A**) injury and orthopaedic evaluation and (**B**) injury and referral.

**Figure 2:**
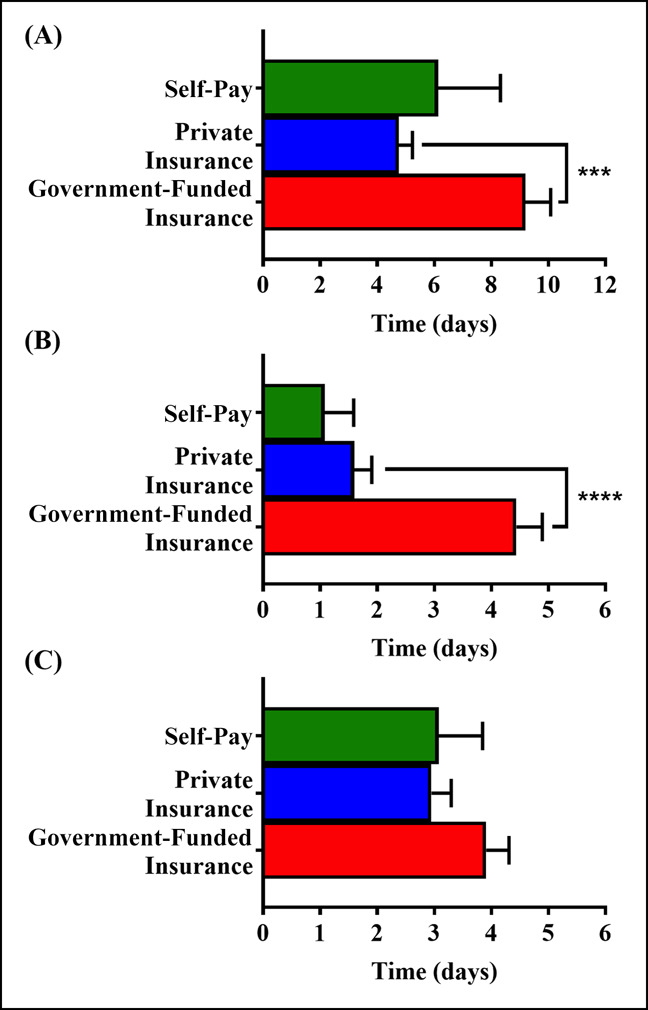
Graph showing the average delay experienced by patients with commercial insurance, government-funded insurance, or self-pay between (**A**) injury and orthopaedic evaluation, (**B**) injury and referral, and (**C**) referral and orthopaedic evaluation. Error bars represent standard error of the mean. ***P ≤ 0.001; ****P ≤ 0.0001.

The delays associated with different insurance groups are listed in Table 3, and the distribution is represented in Figure 1. Analysis of variance results in Table 3 demonstrate a significant difference in the time between injury and orthopaedic evaluation as well as injury and referral. Furthermore, government insurance patients experienced significantly higher time between injury and orthopaedic evaluation (Figure 2) as well as injury and referral (Figure 2) compared with commercial insurance patients. The average time between referral and orthopaedic evaluation for patients relying on government-funded, commercial, and self-pay was not significantly different. Thus, the delay between injury and referral is the primary reason for the delay in orthopaedic evaluation experienced by patients with government-funded insurance. A significant difference was not found (*P* > 0.05) between low-income and high-income patients for time between injury and referral, referral and orthopaedic evaluation, or injury and orthopaedic evaluation.

**Table 3:** Mean and Median Delay and Range in Receiving Care Experienced by Government-Funded, Commercial Insurance, and Self-pay Patients

Factors	Government-funded Insurance	Commercial Insurance	Self-pay	*P* Value
Injury to referral				
Mean (±SD)	4.4 ± 6.9	1.6 ± 3.4	1.1 ± 1.8	<0.001
Median	3	0	0	
Range	0-56	0-19	0-6	
Referral to orthopaedic evaluation				
Mean (±SD)	3.9 ± 6.2	2.9 ± 3.8	3.1 ± 2.8	>0.05
Median	2	1	3	
Range	0-25	0-22	0-32	
Injury to orthopaedic evaluation				
Mean (±SD)	9.2 ± 14.0	4.7 ± 5.8	6.1 ± 8.5	0.001
Median	6	3	3	
Range	0-73	0-32	0-35	

A negative binomial regression was performed to confirm these results and establish a correlation between insurance status and delays experienced by patients with government-funded insurance. The income level was not found to have a notable effect on delays at any level. However, a significant association between the insurance type and time between injury and referral (χ^2^ = 27.96, *P* < 0.001) and injury and orthopaedic evaluation (χ^2^ = 23.81, *P* < 0.001) was confirmed. Specifically, patients with government-funded insurance experienced significantly longer time between injury and referral compared with commercial insurance patients (*P* < 0.001, odds ratio = 2.78, 95% CI = 1.86 to 4.14). This delay had a direct impact on the time delay between injury and orthopaedic evaluation, where these government-funded insurance patients experienced significantly higher delays over patients with commercial insurance (*P* < 0.001, odds ratio = 1.82, 95% CI = 1.42 to 2.33). Patients who self-payed did not experience significantly longer times compared with commercial insurance patients in either of the time intervals (*P* > 0.05). Insurance status was not found to have an influence on the time between referral and evaluation, which was similar for all three insurance groups.

## Discussion

Healthcare disparities among pediatric fracture patients have been established within the orthopaedic literature. Pediatric orthopaedic patients with government-funded health insurance experience longer periods of time before receiving definitive treatment compared with commercially-insured patients at a national level.^1,2,3,4,5,6,9,19,20,21,22,23,24^ The current literature successfully identifies these issuess but has been unable to determine why the issue exists or provide solutions that generate meaningful change. Our study attempts to localize this issue based on the timeline of care. Understanding the timeline may lead to solutions that generate meaningful change. Other studies report notable differences in the type of medical treatment received based on insurance type; government-insured patients are less likely to secure a follow-up outpatient visit, resulting in a higher likelihood of returning to the emergency department for the same diagnosis.^7-9,19^

Orthopaedic literature has yet to establish a standard for appropriate timing of definitive fracture care for the pediatric population. The biology of young bone decreases the possibility of malunion caused by delays because of its inherent ability to overcome deformity with growth; however, specific patterns of deformity show lower ability to remodel and can cause permanent functional impairment. Despite being well-described, these specific fracture patterns can often be missed because of difficulty in assessing pediatric radiographs.^18^ Furthermore, because young bone tends to heal quickly, the window in which correction can take place is limited. Any delay in definitive orthopaedic care may negatively affect the ability of the treating physician to obtain acceptable fracture reduction. Thus, timely intervention is critical in this patient population, and the goal of our study is to identify pediatric fracture patients who are at an increased risk and further localize the issue based on the timeline of care; injury to referral versus referral to initial orthopedic evaluation. Here, we have identified patients relying on government-funded insurance, such as Medicaid, to be at a greater risk compared with patients relying on commercial insurance or self-pay. This risk has been borne out in previous literature; however, this study further characterized this risk based on timeline.^1,2,3,4,5,6,12,19-21,26,27^ The delay between injury and referral for patients with government-funded insurance was 2.8 times higher than patients with commercial insurance. This delay directly affects the time to orthopaedic evaluation, where the average wait time, for these patients, is almost twice as long at 9.2 days over commercial insurance patients.

Our findings are consistent with previous studies that associate government-based insurance with increased challenges in receiving timely pediatric fracture treatment.^1,2,3,4,5,6,9,19,20,25^ Importantly, our study identifies a specific time interval in which this delay is occurring, providing meaningful insight into how this disparity can be addressed. Delays experienced by these patients are directly associated with the insurance type and the ability of these patients to obtain orthopaedic referral. The impact of these delays on the clinical outcome is unclear. In addition, these delays are not the only hurdles faced by these patients; inability to obtain appointments with specialty care providers including, orthopaedic surgeons, remains a major concern.^8,26^ Winzia et al,^26^ in their survey of six states, including Texas, found 46% of all the orthopaedic surgery sport medicine specialists who were contacted accept Medicaid as insurance and only 27.1% of offices scheduled an appointment for a patient with Medicaid. These barriers faced by government-funded insurance patients have been attributed to mandated primary care physician referral to see a specialist, low reimbursement, longer payment periods, tedious paperwork, and increased complexity.^26^ In addition, our data demonstrated a notable correlation between insurance status and socioeconomic status; patients residing in zip codes with lower than median income being more likely to rely on government-funded insurance. Furthermore, the Hispanic population in our study was found to be more likely to reside in lower income zip codes (1.5 times) and rely on government-funded insurance (2.5 times) compared with non-Hispanic patients. These demographic factors, which are associated with several adversities, including reliance on public transportation, financial- or work-related obligations, and perceived medical necessity of orthopaedic treatment may also contribute to these delays in care.

Limitations of our study include the retrospective design. Our data were retrieved from chart review, which is inherently associated with certain limitations, such as variable data entry. Furthermore, our study only included patients seen at our outpatient clinic and, as a result, are only representative of our specific subset of patients and the timeline of encounters because they occurred at our facility. Our data also exclude fractures that required urgent or emergent manipulation because these patients have likely already received definitive treatment from an orthopaedic specialist comfortable with the fracture management. Limiting our data to fracture that do not require urgent or emergent manipulation further specifies our research to patients appropriately managed in on an outpatient basis but does not necessarily exclude fractures that require surgical management. Our study design does not allow for analysis of underlying factors, such as other institutions referral protocols, availability of referring providers, or other patient factors that may delay care, but is different from previous studies in that it shows that the delay in care is occurring within a specific time interval, injury to referral. In addition, our institution accepts both commercial and government-funded insurance, which may result in an increased influx of Medicaid patients, who may have failed to obtain appointments with other commercial practice specialists at our clinic. In addition, although our results provide us an index for the assessment of any future policy changes within our institution, our study does not allow us to investigate and comment on specific influencers that caused these notable delays in care in patients with government-funded insurance. Finally, the impact of race was not accounted for in this study. Our patient population primarily comprised Caucasians and Hispanics, which is also representative of our county and the surrounding 15 counties.^27^ The incorporation of several races into two ethnic groups has the potential to introduce bias in comparisons. Race is often associated with socioeconomic status, a factor that is known to be closely linked with reliance on public insurance, which could not be analyzed in this study.

In conclusion, pediatric orthopaedics patients with government-funded health insurance are at an increased risk of experiencing greater delays in definitive fracture care compared with patients relying on commercial insurance or self-pay patients. This delay is confined to the time between injury and orthopaedic referral, suggesting difficulty in obtaining orthopaedic referrals for government-funded patients. We also note a strong association between socioeconomic status and insurance type, with patients in zip codes with lower than median income more likely to rely on government-funded insurance. Within our community, the Hispanic population was found to be more likely to reside in lower income zip codes and rely on government-funded insurance. However, no notable difference was found at any time interval when patients were stratified by socioeconomic status or ethnicity. Localizing this issue within our practice system helps define its boundaries and provide insight to improve access to healthcare and clinic efficiency.
